# Advances in Computational Immunology

**DOI:** 10.1155/2015/170920

**Published:** 2015-12-30

**Authors:** Francesco Pappalardo, Vladimir Brusic, Marzio Pennisi, Guanglan Zhang

**Affiliations:** ^1^Department of Drug Sciences, University of Catania, 95100 Catania, Italy; ^2^Nazarbayev University, Astana 010000, Kazakhstan; ^3^Department of Mathematics and Computer Science, University of Catania, 95100 Catania, Italy; ^4^Boston University, Boston, MA 02201-2021, USA

Computational immunology and immunological bioinformatics are firm and quickly growing research fields. Whereas the former aims to develop mathematical and/or computational methods to study the dynamics of cellular and molecular entities during the immune response [1–4], the latter focuses on proposing methods to investigate big genomic and proteomic immunological-related datasets and predict new knowledge mainly by statistical inference and machine learning algorithms.

The glut of data produced by high-throughput instrumentation, notably genomics, transcriptomics, epigenetics, and proteomics methods, requires computational tools for acquisition, storage, and analysis of immunological data.

The exploitation of such a huge amount of immunological data usually requires its conversion into computational problems, their solution using mathematical and computational approaches, and then the translation of the obtained results into immunologically meaningful interpretations.

In this special issue, we take an interest from mathematicians, bioinformaticians, computational scientists, and engineers together with experimental immunologists to present and discuss latest developments in different subareas of computational immunology, ranging from databases applications to computational vaccine design, modelling, and simulation and their application to basic and clinical immunology.

The review from N. Sepúlveda et al. calls attention to serology data in conjunction with mathematical modelling in providing a powerful approach to inform on malaria transmission intensity and putative changes over time. Their conclusions show that an interesting idea with public health potential is to use a panel of multidisease antibodies that can be instrumental to know what the infectious agents are in circulation in a given population and their putative dynamics. This idea has not been tested in practice, but definitely will require the extension of classical mathematical models to fully account the immunological interaction between different diseases.

In their paper W. Schreiner and colleagues illustrate that molecular dynamics was used to simulate large molecules of the immune system (major histocompatibility complexes, T-cell receptors, and coreceptors). To characterize the relative orientation and movements of domains local coordinate systems (based on principal component analysis) were generated and directional cosines and Euler angles computed. As a most interesting result, they found that the presence of the coreceptor seems to influence the dynamics within the protein complex, in particular the relative movements of the two *α*-helices, G*α*1 and G*α*2.

It is assessed that the application of personalized medicine requires integration of different data to determine each patient's unique * *clinical constitution. The automated analysis of medical data is a growing field where different machine learning * *techniques are used to minimize the time consuming task of manual analysis.

In the paper contributed by C.-M. Svensson et al., the authors investigate the interobserver variability of image data comprising fluorescently stained circulating tumor cells and its effect on the performance of two automated classifiers, a random forest and a support vector machine. They found that uncertainty in annotation between observers limited the performance of the automated classifiers, especially when it was included in the test set on which classifier performance was measured.

Therapeutic protein products (TPP) have been widely used to treat a variety of human diseases, including cancer, hemophilia, and autoimmune diseases. However, TPP can induce unwanted immune responses that can impact both drug efficacy and patient safety. The presence of aggregates is of particular concern as they have been implicated in inducing both T-cell independent and T-cell dependent immune responses. L. Yin and collaborators used mathematical modelling to evaluate several mechanisms through which aggregates of TPP could contribute to the development of immunogenicity. Their computational analyses suggest that aggregates are unlikely to induce T-cell independent antibody responses through BCR cross-linking. In contrast, aggregates could contribute to immunogenicity via the T-cell dependent pathway by inducing the presentation of high affinity epitopes that may not be present in nonaggregated TPP and/or by enhancing danger signal to maturate dendritic cells and activate T-cells.

A. K. Irin et al. investigate computational modelling approaches on epigenetic factors in neurodegenerative and autoimmune diseases and their mechanistic analysis. The authors examine the major milestones in epigenetics research in the context of diseases and various computational approaches developed in the last decades to unravel new epigenetic modifications. However, there are limited studies that systematically link genetic and epigenetic alterations of DNA to the aetiology of diseases, they said. In this work, A. K. Irin and coauthors show how disease-related epigenetic knowledge can be systematically captured and integrated with heterogeneous information into a functional context using Biological Expression Language (BEL). This novel methodology, based on BEL, enables the integration of epigenetic modifications such as DNA methylation or acetylation of histones into a specific disease network.

In the paper by G. Bocharov et al., the authors show how the modelling approaches can be implemented to address diverse aspects of immune system functioning under normal conditions and in response to LCMV and, importantly, make quantitative predictions of the outcomes of immune system perturbations. This may highlight that data-driven applications of meaningful mathematical models in infection biology remain a challenge.

MHC *α*-helices form the antigen-binding cleft and are of particular interest for immunological reactions. To monitor these helices in molecular dynamics simulations, the paper contributed by R. Ribarics et al. applied a parsimonious fragment-fitting method to trace the axes of the *α*-helices. Each resulting axis was fitted by polynomials in a least-squares sense and the curvature integral was computed. To find the appropriate polynomial degree, the method was tested on two artificially modelled helices, one performing a bending and another a hinge movement. They found that second-order polynomials retrieve predefined parameters of helical motion with minimal relative error.

There are at present few tools available to assist with the determination and analysis of B-cell lineage trees from next-generation sequencing data. The paper from W. D Lees and A. J. Shepherd presents two utilities that support automated large-scale analysis and the creation of publication-quality results. The tools are available on the web and are also available for download so that they can be integrated into an automated pipeline. These utilities can be used with any suitable phylogenetic inference method and with any antibody germline library and hence are species-independent.

Vaccination is historically one of the most important medical interventions for the prevention of infectious disease. Previously, vaccines were typically made of rather crude mixtures of inactivated or attenuated causative agents. However, over the last 10–20 years, several important technological and computational advances have enabled major progress in the discovery and design of potently immunogenic recombinant protein vaccine antigens. L. Liljeroos and colleagues discuss three key breakthrough approaches that have potentiated structural and computational vaccine design. They illustrate the growing power of combining sequencing, structural, and computational approaches and discuss how this may drive the design of novel immunogens suitable for future vaccines urgently needed to increase the global prevention of infectious disease.

MIrExpress is a new database which takes advantage of the information theory, as well as the Pearson linear correlation method, to measure the linear correlation, nonlinear correlation, and their hybrid of cell-specific gene coexpressions in immune cells. In the work from J. Wang et al., the authors describe this database that totally includes 16 human cell groups, involving 20,283 human genes. The expression data and the calculated correlation results from the database are interactively accessible on the web page and can be implemented for other related applications and researches.

Publically available influenza data are a valuable resource for computational analyses with applications in vaccine design. Similarly, existing bioinformatics tools provide the means for extraction of information and new knowledge. However, to utilize the full potential of these resources, data preprocessing must be performed and analytical tools must be carefully combined into well-defined workflows. C. Simon et al. describe FluKB, a knowledge-based system focusing on data and analytical tools for influenza vaccine discovery. The main goal of FluKB is to provide access to curated influenza sequence and epitope data and enhance the analysis of influenza sequence diversity and the analysis of targets of immune responses. FluKB consists of more than 400,000 influenza protein sequences, known epitope data (357 verified T-cell epitopes, 685 HLA binders, and 16 naturally processed MHC ligands), and a collection of 28 influenza antibodies and their structurally defined B-cell epitopes.

*Francesco Pappalardo* *Francesco Pappalardo* *Vladimir Brusic* *Vladimir Brusic* *Marzio Pennisi* *Marzio Pennisi* *Guanglan Zhang* *Guanglan Zhang*
